# Meningo-cortical manifestations of myelin oligodendrocyte glycoprotein antibody-associated disease: Review of a novel clinico-radiographic spectrum

**DOI:** 10.3389/fneur.2022.1044642

**Published:** 2022-10-20

**Authors:** Adrian Budhram, Ario Mirian, Manas Sharma

**Affiliations:** ^1^Department of Clinical Neurological Sciences, Western University, London Health Sciences Centre, London, ON, Canada; ^2^Department of Pathology and Laboratory Medicine, Western University, London Health Sciences Centre, London, ON, Canada; ^3^Department of Medical Imaging, Western University, London Health Sciences Centre, London, ON, Canada

**Keywords:** MOG, FLAMES, cerebral cortical encephalitis, meningitis, autoimmune neurology, neuroimmunology, MOGAD

## Abstract

Myelin oligodendrocyte glycoprotein antibody-associated disease (MOGAD) is an inflammatory demyelinating disease that is distinct from multiple sclerosis. Initial manifestations of MOGAD that were reported in the literature included optic neuritis, myelitis, brainstem demyelination and encephalitis, with emphasis placed on acute disseminated encephalomyelitis (ADEM) as the primary encephalitic presentation. In 2017, however, Ogawa et al. described four patients with seizures, unilateral cortical hyperintensities on brain magnetic resonance imaging T2-fluid-attenuated inversion recovery sequences, and anti-MOG positivity, indicating a potentially novel form of encephalitis in MOGAD. In 2019, we systematically reviewed the literature to better characterize this unique syndrome, which we referred to as unilateral cortical FLAIR-hyperintense Lesions in Anti-MOG-associated Encephalitis with Seizures (FLAMES). Subsequently, anti-MOG positivity in patients with a variety of cortical and meningeal disease presentations has been reported, indicating a broader spectrum of meningo-cortical manifestations in MOGAD that we review herein.

## Introduction

**Figure 1:**
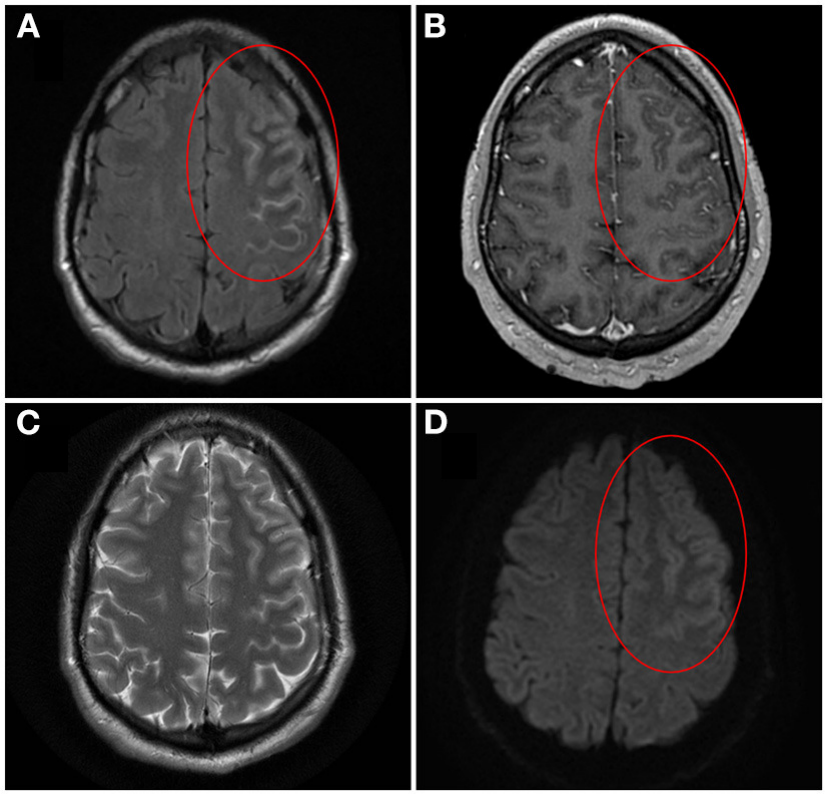
Brain magnetic resonance imaging of unilateral cortical FLAIR-hyperintense Lesion in Anti-MOG-associated Encephalitis with Seizures (FLAMES)/Unilateral cerebral cortical encephalitis (UCCE). On axial T2-weighted fluid-attenuated inversion recovery (T2-FLAIR) image pre-gadolinium, cortical swelling and hyperintensity of both the left frontal cortex and adjacent sulci is seen, with hypointensity of the adjacent juxtacortical white matter [**(A)** circle]. On axial T1-weighted image post-gadolinium, corresponding leptomeningeal enhancement is also seen [**(B)** circle]. The cortical hyperintensity is not well visualized on axial T2-weighted image **(C)**. On axial diffusion-weighted image there is brightness of the cortex with sparing of the subarachnoid space [**(D)** circle]. Corresponding subtle darkness on apparent diffusion coefficient map was seen, compatible with true diffusion restriction (not shown). Image adapted and re-used with permission from Springer Nature: Budhram A, Mirian A, Le C, Hosseini-Moghaddam SM, Sharma M, Nicolle MW. Unilateral cortical FLAIR-hyperintense Lesions in Anti-MOG-associated Encephalitis with Seizures (FLAMES): characterization of a distinct clinico-radiographic syndrome. *J Neurol*. (2019) 266(10):2481–7.

Over the last decade, antibodies against myelin oligodendrocyte glycoprotein (MOG) have emerged as a biomarker of inflammatory demyelinating disease that is distinct from multiple sclerosis (1–7). Classical manifestations of MOG antibody-associated disease (MOGAD) that were initially described in the literature included optic neuritis, myelitis, brainstem demyelination and encephalitis. In particular, mention of encephalitis in MOGAD has historically been in reference to acute disseminated encephalomyelitis (ADEM), which is a multifocal inflammatory demyelinating disease that presents with encephalopathy and large T2-hyperintense lesions predominantly involving the cerebral white matter (8). The focus on ADEM as the primary encephalitic manifestation of MOGAD was highlighted in early recommendations on anti-MOG testing and diagnosis, which did not emphasize any other cerebral disease presentations (9, 10). In 2017, however, Ogawa et al. reported four patients who they described as having MOG antibody-positive, benign, unilateral, cerebral cortical encephalitis with epilepsy, indicating a novel phenotype of MOGAD (11). All four patients had seizures, unilateral cortical hyperintensities on brain magnetic resonance imaging (MRI) T2-fluid-attenuated inversion recovery (T2-FLAIR) sequences, and anti-MOG positivity (11). In 2019, we systematically reviewed the literature to better characterize this unique syndrome and proposed the term unilateral cortical FLAIR-hyperintense Lesions in Anti-MOG-associated Encephalitis with Seizures (FLAMES; Figure 1), which has since been adopted in the literature (12–18). Although initially reported to be a unilateral cortical encephalitis, we noted the presence of bilateral cortical involvement and possible meningeal inflammation in a subset of cases that was suggestive of a broader disease spectrum. In recent years, this possibility has been supported by the dramatic rise in reports of anti-MOG-positive patients with a variety of cortical and meningeal presentations (19–24). We herein review these meningo-cortical manifestations of MOGAD, with the aim of facilitating their prompt recognition when encountered in clinical practice.

## Unilateral cortical FLAMES/UCCE

Unilateral cortical FLAMES, also referred to as unilateral cerebral cortical encephalitis (UCCE), was first highlighted as a potentially novel manifestation of MOGAD by Ogawa et al. in 2017 (11, 12, 22, 25). While patients with compatible disease presentations had been published prior to this seminal work, they were not recognized as potentially harboring a unique MOGAD phenotype. In 2015, Kim et al. described a 34-year-old male with headache, fever, and fluctuating level of consciousness ascribed to seizure activity, along with unilateral cortical T2-FLAIR hyperintensity (26). Antibodies against the N-methyl-D-aspartate receptor (NMDAR) were detected, and he was diagnosed with anti-NMDAR encephalitis. No testing for anti-MOG was reported; however, his neuroimaging findings, clinical presentation, and rapid improvement with only a relatively short course of corticosteroids was highly compatible with unilateral cortical FLAMES/UCCE, which has since been reported to co-exist with anti-NMDAR in a minority of cases (20, 21). Subsequently, in 2016, Numa et al. described a 37-year-old female with a remote history of ADEM who presented with headache, fever and right eye vision loss, followed by abnormal sensation in the right extremities (27). Neuroimaging revealed findings of right optic neuritis as well as the development of left frontal and temporal cortical T2-FLAIR hyperintensities that were suggestive of unilateral cortical FLAMES/UCCE, but that were interpreted at the time as multiphasic ADEM. Fortunately, following its emergence in the literature as a novel manifestation of MOGAD, cases of unilateral cortical FLAMES/UCCE have increasingly been recognized. At present it is the most well-characterized entity within the spectrum of meningo-cortical manifestations that have been observed in MOGAD, with distinct neuroimaging findings, clinical features, cerebrospinal fluid (CSF) abnormalities and treatment considerations that are summarized in Table 1 and discussed below.

**Table 1:** Key features of unilateral cortical FLAMES/UCCE.

	**Description of typical findings**	**Additional considerations**
**MRI findings**	Unilateral cortical T2-FLAIR hyperintensity is observed that can affect any lobe of the brain	The frontal and parietal lobes are most often involved, while occipital lobe involvement is least common
	Corresponding hyperintensity on T2WI may be minimal or absent	Progression to bilateral cortical T2-FLAIR hyperintensity, as well as sulcal T2-FLAIR hyperintensity and/or overlying leptomeningeal enhancement, is reported in a subset of patients and supports a broader disease spectrum
	T2-FLAIR hyperintensity of adjacent juxtacortical white matter is characteristically absent at presentation, but may be seen with other cerebral manifestations of MOGAD including ADEM	T2-FLAIR hypointensity of adjacent juxtacortical white matter may be a supportive diagnostic feature
**Clinical symptoms**	Seizures are most prominent clinical manifestation and are observed in large majority of patients	Seizures are focal-onset, most often present as motor seizures, ictal aphasia or somatosensory symptoms, and frequently progress to bilateral tonic-clonic seizures
	Headache, fever, cortical symptoms referable to the lesion location, and other features of encephalopathy are also characteristic manifestations	Cortical symptoms referable to the lesion that are most commonly reported include aphasia and hemiparesis, although depending on the lesion location symptoms such as hemianopsia may occur
	Patients may have other attacks compatible with MOGAD prior to, concurrent with, or after unilateral cortical FLAMES/UCCE	Headache may have features suggestive of increased intracranial pressure
		Prior to diagnosing cerebral dysfunction in the absence of seizures, consider prolonged EEG to assess for the possibility that symptoms are ictal/post-ictal phenomena
**CSF evaluation**	Pleocytosis is observed that can be >100 WBC/μl but is usually < 1000 WBC/μl	Approximately 10% of patients have been reported to have normal CSF WBC count
	Opening pressure may be elevated and should be measured at time of lumbar puncture	Less than 20% of patients have been reported to have CSF-specific oligoclonal bands
	A minority of patients have been reported to have co-existent anti-NMDAR, for which testing should be considered and detection is optimal in CSF	While testing for anti-MOG in serum is generally recommended, testing of stored CSF may be helpful if serum at time of attack is not available
**Treatment**	Excellent response to corticosteroids is observed, typically with resolution of clinical symptoms and neuroimaging findings	Although treatment with corticosteroids is generally recommended, cases of resolution without immunotherapy have also been reported
	Anti-seizure medications are commonly administered once seizures are identified but their necessity, particularly long-term, is uncertain	Optimal maintenance immunotherapy in patients with relapsing disease is unclear

ADEM, acute disseminated encephalomyelitis; CSF, cerebrospinal fluid; DWI, diffusion-weighted imaging; EEG, electroencephalography; MOG, myelin oligodendrocyte glycoprotein; MOGAD, MOG antibody-associated disease; MRI, magnetic resonance imaging; NMDAR, N-methyl-D-aspartate receptor; T2-FLAIR, T2-fluid-attenuated inversion recovery; T2WI, T2-weighted imaging; WBC, white blood cell.

### Neuroimaging findings in unilateral cortical FLAMES/UCCE

Patients with this syndrome have cortical hyperintensity that can affect any lobe of the brain (12, 21, 28, 29). The frontal and parietal lobes are most often involved, while occipital lobe involvement is least common (21). As the name suggests, unilateral involvement is typically observed, although development of bilateral cortical encephalitis with anti-MOG positivity has been described in a small number of patients (discussed later) (12, 20, 21, 23, 30, 31). The cortical hyperintensity is typically most evident on T2-FLAIR sequences, so these should be carefully reviewed for subtle abnormality that may otherwise be missed (11, 12, 32). In contrast, corresponding hyperintensity on T2-weighted imaging (T2WI) may be minimal or absent (11, 12). Classically, the hyperintensity is only cortical at presentation, without involvement of the adjacent juxtacortical white matter (Figure 1) (12, 22). However, T2-FLAIR-hyperintense lesions involving both the cortex and adjacent juxtacortical white matter may be observed with other cerebral presentations of MOGAD including ADEM, and so their presence still warrants consideration of anti-MOG testing in the appropriate clinical context. Attention has recently been drawn to T2-FLAIR hypointensity of the adjacent juxtacortical white matter (Figure 1), and the presence of this finding alongside cortical T2-FLAIR hyperintensity may provide further supportive evidence of unilateral cortical FLAMES/UCCE (12, 22). Hyperperfusion of the cortical lesions can be seen on brain perfusion scans such as single photon emission computed tomography (SPECT), although this testing may not be routinely performed in clinical practice (11, 21). Additional neuroimaging findings that have been observed in a subset of cases include sulcal T2-FLAIR hyperintensity and leptomeningeal enhancement, which are suggestive of leptomeningeal inflammation (12). A small number of patients with leptomeningeal enhancement and symptoms of meningitis (e.g. headache, fever) in the absence of symptoms of encephalitis (e.g. seizures, cortical symptoms, other features of encephalopathy) have been described, indicating a broader spectrum of meningo-cortical manifestations that includes predominantly meningeal disease (discussed later) (33, 34). Similar neuroimaging features can be seen in other disease processes involving the cortex (e.g. Creutzfeldt-Jakob disease, mitochondrial disease, hypoxia, metabolic disturbance, seizure-related change) or the subarachnoid space (e.g. subarachnoid hemorrhage, infectious or carcinomatous meningitis), so alternative diagnoses relevant to the patient presentation should still be thoroughly considered when such imaging abnormalities are encountered (11, 12, 35–39). However, lesions with a typical appearance for unilateral cortical FLAMES/UCCE on MRI should prompt testing for anti-MOG in the appropriate clinical context.

### Clinical features of unilateral cortical FLAMES/UCCE

In line with other autoimmune encephalitides, patients with this syndrome typically present subacutely (8, 12). While acute stroke-like presentations have been reported, the history in such cases should be carefully reviewed for symptoms in the preceding days to weeks that may otherwise go unrecognized, such as headache, fever, or intermittent spells suggestive of seizures (12, 40). Seizures are the most prominent clinical manifestation of unilateral cortical FLAMES/UCCE, and occur in the large majority of patients. Our previous review of cases published in the literature found that seizures were reported in 85%, and a recent series of 39 Japanese patients noted that nearly all (97%) presented with seizures (12, 22). They are focal-onset, most often present as motor seizures, ictal aphasia or somatosensory symptoms, and have been reported to progress to bilateral tonic-clonic seizures in as many as 77% (12, 32). Seizure semiology commonly corresponds to lesion location, supporting their epileptogenicity. In addition to seizures, characteristic disease manifestations include headache, fever, cortical symptoms referable to the lesion location, and other features of encephalopathy (12, 21, 28, 29). Headache can be severe, and may have features of high intracranial pressure (12). Cortical symptoms referable to the lesion that are most commonly reported include aphasia and hemiparesis, although depending on the lesion location symptoms such as hemianopsia may occur (28, 41). Other features of encephalopathy that may be observed include altered mental status and behavioral change (11, 12, 28). Importantly, in patients presenting with such symptoms of cerebral dysfunction in the absence of recognized seizures, the possibility that their presentation may represent an ictal/post-ictal phenomenon should be considered, in which case prolonged electroencephalography can be helpful to look for electroencephalographic evidence of seizures that may otherwise go undiagnosed (12, 42). Although the term “epilepsy” was initially used to describe these patients, an enduring predisposition to seizures following treatment does not generally occur. For this reason, use of the term “seizures” rather than “epilepsy” more appropriately reflects their typically acute symptomatic nature (11, 12, 43).

### CSF abnormalities in unilateral cortical FLAMES/UCCE

In patients suspected of having this syndrome, a number of CSF abnormalities have been described. As mentioned previously, patients with unilateral cortical FLAMES/UCCE can present with severe headache that has features of high intracranial pressure. In line with this, CSF opening pressure may be elevated and should be measured at time of lumbar puncture (12, 20). CSF white blood cell (WBC) count elevation has been reported in as many as 90% of patients, with pleocytosis that may be in excess of 100 WBC/μl but is usually less than 1000 WBC/μl (12, 20, 22). CSF-specific oligoclonal bands have been reported in less than 20% of patients, which stands in contrast to nearly 90% of patients with MS (12, 20, 44). However, unlike other manifestations of MOGAD such as optic neuritis or myelitis, MS is less likely to be a leading diagnostic consideration in patients with unilateral cortical FLAMES/UCCE because, although cortical presentations of MS with focal-onset seizures have been described, they are rare and lack the characteristic neuroimaging features (45, 46). A neuro-inflammatory disease that may more closely resemble unilateral cortical FLAMES/UCCE is anti-NMDAR encephalitis given its subacute onset, frequent viral prodrome, and propensity to cause seizures (8, 47). Intriguingly, a minority of patients with unilateral cortical FLAMES/UCCE have been reported to harbor co-existent anti-NMDAR; while the uniform detection of anti-MOG in patients with this clinico-radiographic syndrome suggests it is the more relevant disease biomarker, CSF testing of anti-NMDAR should therefore be considered in all cases and may be helpful to inform the potential for phenotypic overlap, role of malignancy screening, treatment considerations, and prognosis in dual-positive patients (20, 21, 48). Although testing for anti-MOG in serum is generally recommended and has overall higher clinical sensitivity for MOGAD compared to CSF, testing of stored CSF may be helpful in cases of unilateral cortical FLAMES/UCCE when serum from the time of attack is not available (40, 49, 50).

### Treatment of unilateral cortical FLAMES/UCCE

In keeping with other manifestations of MOGAD, patients with unilateral cortical FLAMES/UCCE generally have excellent response to corticosteroids (12, 22). Anti-seizure medications are commonly administered once seizures are identified, but seizures typically resolve after corticosteroid treatment. The necessity of anti-seizure medication is thus uncertain, particularly outside the acute symptomatic phase (12). While treatment with corticosteroids is generally recommended, cases of resolution without immunotherapy have also been reported (40, 51). Expectedly, patients can have other attacks compatible with MOGAD prior to, concurrent with, or after unilateral cortical FLAMES/UCCE (12). Maintenance immunotherapy should be considered in patients with MOGAD who have relapsing disease, although the optimal long-term treatment approach remains unclear (52–55).

### Pathologic findings in unilateral cortical FLAMES/UCCE

To date, pathologic data remains sparse among patients with this syndrome. In 2018, Ikeda et al. described a 29-year-old female who presented with unilateral cortical FLAMES/UCCE and underwent brain biopsy of the involved right parietal gyrus while symptomatic, prior to immunotherapy (56). The subarachnoid space and brain parenchyma revealed mild lymphocytic infiltration and perivascular lymphocyte cuffing without distinct demyelination, although the authors questioned whether the biopsy findings could reflect a very early stage of demyelinating disease. In 2019, Patterson et al. described a 39-year-old female with unilateral cortical FLAMES/UCCE who underwent brain biopsy of the involved left parietal lobe and dura while symptomatic, prior to immunotherapy (57). This showed interstitial and perivascular lymphocytic infiltrates, without clear evidence of demyelination. In 2020, Takai et al. reviewed 11 brain biopsies of patients with MOGAD, two of whom were classified as having cortical encephalitis (58). One was previously described (56), while the other was a 15-year-old female who underwent biopsy of a left temporal lobe lesion after initiation of corticosteroids. Of note, this patient had T2-FLAIR hyperintensity involving both the cortex and adjacent juxta-cortical white matter. In addition to perivascular lymphocytic infiltrates, subpial and perivenous demyelination with cortical encephalitis was observed. A similar pattern of demyelination was observed in one patient with ADEM, leading the authors to hypothesize that ADEM and cortical presentations of MOGAD may have a shared underlying pathology (58).

Differing reports regarding demyelination in unilateral cortical FLAMES/UCCE may relate to one or more factors. It is possible that patchy demyelination could be missed depending on the biopsy location, leading to sampling variability. Variable reports of demyelination could also reflect different stages of disease, depending on timing of biopsy. Finally, it bears emphasis that typically, neuroimaging of unilateral cortical FLAMES/UCCE at presentation shows cortical T2-FLAIR hyperintensity without hyperintensity of the adjacent juxtacortical white matter, rather than T2-FLAIR hyperintensity of both (12, 22). Labeling patients with strictly cortical T2-FLAIR hyperintense lesions and patients with T2-FLAIR hyperintense lesions involving the cortex and adjacent juxtacortical white matter as both having “cortical encephalitis” could hinder the discovery of pathologic findings that may potentially distinguish between meningo-cortical manifestations of MOGAD and other manifestations such as ADEM, a possibility that should be considered in future pathologic analyses.

## Other meningo-cortical manifestations of MOGAD

### Bilateral cortical manifestations, including BFCCE/BPCLI

Although unilateral cortical FLAMES/UCCE is classically an affliction of one hemisphere, progression to bilateral cortical involvement has been reported in a subset of cases (12). Among patients with anti-MOG positivity and bilateral cortical involvement, the frontal lobes are most commonly involved (21). An exemplary case of this was provided by Fujimori et al. who in 2017 described a 46-year-old male with cortical T2-FLAIR hyperintensity of the left medial frontal cortex and a focal motor seizure of the right leg that secondarily generalized (30). While disease was initially limited to one hemisphere, the patient went on to develop more extensive bilateral medial frontal cortical T2-FLAIR hyperintensities as well as paraparesis. He was treated empirically with high-dose corticosteroids for suspected autoimmune encephalitis and had good response. Biopsy of the right cingulate gyrus showed lymphocytic infiltrates without demyelination, with the caveat that it was performed after approximately 1 month of corticosteroids. The patient did well until he developed optic neuritis nearly 2 years later, and subsequent testing of stored serum from the time of his initial encephalitic presentation revealed positivity for anti-MOG. Fujimori et al. later reviewed the literature for similar cases of what they referred to as MOG antibody-associated bilateral medial frontal cerebral cortical encephalitis (BFCCE), and identified six patients (23). Similar to unilateral cortical FLAMES/UCCE, a subset of those with BFCCE were noted to have overlying leptomeningeal enhancement, which has also been referred to in the literature as bilateral parafalcine cortical and leptomeningeal impairment (BPCLI) (31). Other disease features of MOG antibody-associated BFCCE/BPCLI (headache, fever, seizures, cortical symptoms referable to the lesion location in the form of paraparesis, CSF pleocytosis and steroid-responsiveness) closely resemble those observed in unilateral cortical FLAMES/UCCE, suggesting that the two entities exist on a continuum of meningo-cortical manifestations in MOGAD (12, 23, 31). To examine differences in topography, Fujimori et al. superimposed lesions in unilateral cortical FLAMES/UCCE and BFCCE/BPCLI and found that lesions in the former were mostly located in the territories of the middle cerebral arteries, while lesions in the latter were mostly located in the territories of the anterior cerebral arteries (23). Taken together with reports of vessel dilation (e.g. on magnetic resonance angiography) and lesion hyperperfusion (e.g. on SPECT) in patients with cortical presentations of MOGAD, the authors suggested that vascular involvement may influence the location of lesion development, which is a hypothesis that would benefit from further study (14, 21, 23, 59).

### Predominantly meningeal manifestations, including FUEL and MOGAM

A subset of patients with both unilateral and bilateral cortical manifestations of MOGAD have been reported to have overlying leptomeningeal enhancement, indicating possible meningeal inflammation and the potential for broader disease involvement beyond the cortex (12, 23, 33). Intriguingly, at the time of our initial literature review of unilateral cortical FLAMES in 2019, we identified one patient who been reported to have unilateral sulcal T2-FLAIR hyperintensity and leptomeningeal enhancement, without significant cortical T2-FLAIR hyperintensity (12, 60). This patient had no symptoms referable to this finding, which was incidentally identified during evaluation of optic neuritis (60). Subsequently, a review of the Mayo Clinic anti-MOG-positive database identified two patients with unilateral leptomeningeal enhancement and minimal-to-no cortical T2-FLAIR hyperintensity, which was referred to as FLAIR-variable Unilateral Enhancement of the Leptomeninges (FUEL) (34, 61). At the time of leptomeningeal enhancement, both of these patients had symptoms suggestive of meningeal irritation (e.g. headache, fever) without additional features to indicate encephalitis (e.g. seizures, focal cortical symptoms, other features of encephalopathy). These reports indicate that predominantly meningeal involvement falls on the continuum of meningo-cortical manifestations observed in MOGAD (34, 60, 61). This was emphasized in a review of twelve patients with MOG antibody positivity and aseptic meningitis in the absence of brain parenchymal lesions, which the authors referred to as MOG antibody-associated aseptic meningitis (MOGAM) (24). In keeping with meningeal inflammation all patients had CSF pleocytosis, and elevated CSF opening pressure was documented in the four who had this measured. Clinically, all had headache and/or fever, while only one-third had seizures; the clinical symptoms reported in MOGAM were therefore similar to those observed in cortical presentations of MOGAD, but with expected differences in relative frequencies that likely relate to whether involvement of the meninges or cortex predominates (12, 24). Excellent response to corticosteroid was generally observed in patients with MOGAM, further supporting the notion that it exists on a broader spectrum of meningo-cortical manifestations in MOGAD.

## Conclusion

Over the last 5 years, dramatic advancements have been made in the characterization of anti-MOG-positive cortical and meningeal presentations. Variations in these presentations, such as whether neuroimaging abnormalities are unilateral or bilateral, or whether cortical or meningeal involvement predominates, have been described by different groups and led to a plethora of nomenclature that includes unilateral cortical FLAMES, UCCE, BFCCE, BPCLI, FUEL and MOGAM. However, commonalities across their neuroimaging findings, clinical features, CSF parameters and responses to treatment suggest that these entities lie on a continuum of meningo-cortical manifestations in MOGAD. Increased recognition of this novel clinico-radiographic spectrum is essential for accurate patient diagnosis, as well as future studies dedicated to uncovering potentially unique pathophysiologic mechanisms.

## Author contributions

AB planned and drafted the manuscript. AM and MS revised the manuscript for intellectual content. All authors contributed to the article and approved the submitted version.

## Funding

AB reports that he holds the London Health Sciences Centre and London Health Sciences Foundation Chair in Neural Antibody Testing for Neuro-Inflammatory Diseases, and is supported by the Opportunities Fund of the Academic Health Sciences Centre Alternative Funding Plan of the Academic Medical Organization of Southwestern Ontario (AMOSO).

## Conflict of interest

The authors declare that the research was conducted in the absence of any commercial or financial relationships that could be construed as a potential conflict of interest.

## Publisher's note

All claims expressed in this article are solely those of the authors and do not necessarily represent those of their affiliated organizations, or those of the publisher, the editors and the reviewers. Any product that may be evaluated in this article, or claim that may be made by its manufacturer, is not guaranteed or endorsed by the publisher.
